# Supplementary material for: Plasma Clearance of Coagulation Factor VIII and Extension of Its Half-Life for the Therapy of Hemophilia A: A Critical Review of the Current State of Research and Practice
Source: Int J Mol Sci. 2023 May 11;24(10):8584. doi: 10.3390/ijms24108584 (PMC10217895; doi:10.3390/ijms24108584)
Supplement: Supplementary file 1 [file ijms-24-08584-s001.zip › ijms-2376756-supplementary.pdf]

From *Church et al, 2018* [106]		Spiked FVIII Recovery (%)			
	FVIII_Spiked	0.04	0.4	0.9	(IU/mL)
Bay 94-9027	OC	107	92	78	
	CS	107	120	118	
	OC/CS	1.00	0.77	0.66	
FL-FVIII	OC	122	105	100	
	CS	92	102	110	
	OC/CS	1.33	1.03	0.91	
**Bay 94-9027 OC/CS difference relatively FL-VIII OC/CS** (OC/CS_FL-FVIII - OC/CS_Bay 94-9027)		0.33	0.26	0.25	

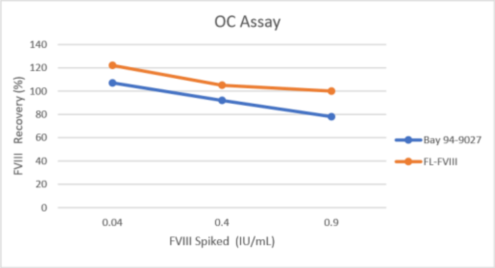

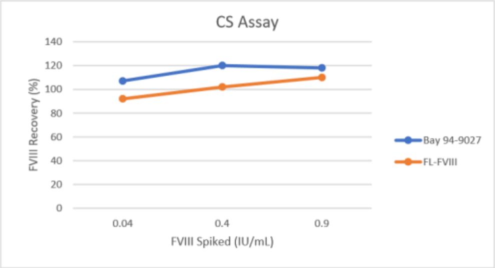

From *Tiefenbacher et al, 2019* [110]		Spiked FVIII Recovery (%)				
	FVIII_Spiked	0.03	0.2	0.6	0.9	(IU/mL)
N8-GP	OC	101.7	95.3	87.7	86.1	
	CS	119.1	130.5	135	132.7	
	OC/CS	0.85	0.73	0.65	0.65	
FL-FVIII	OC	146.1	124.2	114.2	109.1	
	CS	123.5	126	132.5	127.9	
	OC/CS	1.18	0.99	0.86	0.85	
**N8-GP OC/CS difference relatively FL-VIII OC/CS** (OC/CS_FL-FVIII - OC/CS_N8-GP)		0.33	0.26	0.21	0.20	

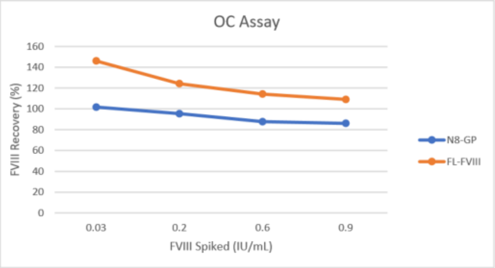

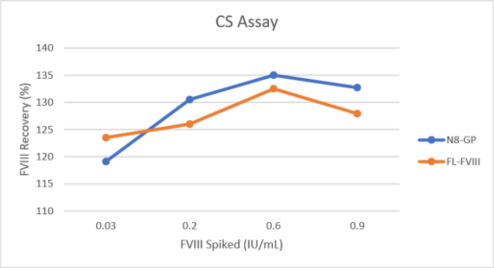

Figure S1. Analysis of plasma samples spiked with selected FVIII variants using OC and CS assays.

The data for the FVIII variants recovery by OC and CS assays were taken from the original papers (*Church et al.* [106] and *Tiefenbacher et al.* [110]) and reprocessed as shown.
